# Inhibitory effect and possible mechanism of a *Pseudomonas* strain QBA5 against gray mold on tomato leaves and fruits caused by *Botrytis cinerea*

**DOI:** 10.1371/journal.pone.0190932

**Published:** 2018-01-10

**Authors:** Pan Gao, Jiaxing Qin, Delong Li, Shanyue Zhou

**Affiliations:** 1 College of Plant Health and Medicine, Qingdao Agricultural University, Qingdao, China; 2 The Key Lab of Integrated Crop Pests Management of Shandong Province, Qingdao Agricultural University, Qingdao, China; Shanghai Jiao Tong University, CHINA

## Abstract

The fungal pathogen *Botrytis cinerea* causes gray mold disease on various hosts, which results in serious economic losses. Over the past several decades, many kinds of fungicides have been used to successfully control the disease. Meanwhile, the uses of fungicides lead to environmental pollution as well as a potential threat to the human health by the chemical residues in tomato fruit. Also, the gray mold disease is difficult to control with fungicides. Therefore, exploring alternative measures such as biological controls could be the best choice to control the disease and alleviate damages caused by fungicides. In this study, we isolated and identified a novel *Pseudomonas* strain termed as QBA5 from healthy tomato plant based on the morphological, biochemical characteristics and molecular detection. The antifungal activity assays revealed that, in the presence of QBA5, conidia germination, germ tube elongation and mycelial growth of *B*. *cinerea* were significantly inhibited. Most importantly, QBA5 exerted a significant preventive effectiveness against gray mold on tomato fruits and plants. The possible mechanism of QBA5 involved in the inhibition of *B*. *cinerea* was investigated. It revealed that the conidia plasma membrane of *B*. *cinerea* was severely damaged by QBA5. Further, four different antifungal compounds in the supernatant of QBA5 were separated by preparative high performance liquid chromatography (PHPLC). Overall, the data indicate that there is a considerable potential for QBA5 to reduce the damage caused by gray mold disease on tomato.

## Introduction

The fungal pathogen *Botrytis cinerea* Pers.:Fri. (teleomorph *Botryotinia fuckeliana* (de Bary) Whetzel) infects plants causing gray mold disease. It is a devastating disease on tomato leading to serious economic losses in many countries worldwide [1, 2, 3, 4]. Under the suitable environmental conditions, especially under high humidity and low temperature, the typical symptoms of this disease can be visualized on tomato fruit and every part of the tomato plant infected by the pathogen [2, 4]. Usually the pathogen is quiescent in the host tissues for a long period of time, hence, the symptoms of this disease appear much later than hosts infection. Consequently, some apparently healthy tomato fruit rot rapidly during the storage period or during the transport to the market. Therefore, the gray mold disease causes damage both before and after tomato harvesting.

Furthermore, the pathogen is very difficult to control for several reasons. First, the pathogen has a wide range of plant hosts, by reports, including more than 200 crop species [4, 5]. Second, the pathogen can survive as mycelia and/or conidia or sclerotia in crop debris in or after the plant’s growth season [4]. Third, gene mutation of the pathogen occurred rapidly and fungicide resistance of the pathogen developed easily in the fields. Over the past several decades, many kinds of fungicides have been used to successfully control the disease. However, evidence demonstrates that isolates of *B*. *cinerea* have developed some specific resistances to several fungicides including dicarboximides, benzimidazole, phenylpyrrole and hydroxyanilide [6, 7, 8, 9, 10]. Worse still, misuse and excessive use of fungicides for years result in environmental pollution, disturbance of natural ecosystems, and pose a potential threat to human health because of the high level of toxic residues of fungicides in tomato fruit [1, 3, 11, 12]. In order to overcome the obstacles resulting from the chemical fungicides, there is an urgent need for alternative measures that could safely and effectively control gray mold disease on tomato. The biological control is undoubtedly the best choice.

In general, microbial biological control agents (BCAs) such as fungi, bacteria and actinomyces can effectively control plant diseases by antibiosis [13, 14, 15], competition for nutrients and niches [16], parasitism [17, 18], volatile organic compounds (VOCs) [1, 19, 20] or induction of plant resistance [21, 22, 23]. It is well demonstrated that some bacterial species of the *Bacillus* and *Pseudomonas* genus could effectively control plant diseases by producing antibiotics [24, 25, 26, 27] or stimulating the host resistance [28, 29]. Most interestingly, some BCAs play their biological control roles in multiple patterns. For instance, *Clonostachys rosea* can control gray mold disease not only by suppressing development and sporulation of *B*. *cinerea* but also by inducing the resistance of tomato plants against *B*. *cinerea* [12, 30, 31].

There is growing evidence that a large number of microorganisms from the phyllosphere and rhizosphere act as antagonists to plant pathogens [32, 33]. With the aim to acquire some antagonists to *B*. *cinerea* in this work, we isolated and detected the antifungal activity of the bacteria strains from healthy tomato leaves. Finally, we isolated and identified a novel *Pseudomonas* strain termed as QBA5 based on its morphology, biochemical characteristics and molecular detection. We examined the inhibitory effects of the QBA5 on *B*. *cinerea* with its cell-free supernatant. Furthermore, we investigated the underlying mechanism of the QBA5 inhibitory effect on *B*. *cinerea*. We also tested the effectiveness of QBA5 on disease severity reduction using detached tomato leaves and fruit. Overall, the data presented in this work show that QBA5 can function as an efficient BCA to reduce the severity of gray mold disease caused by *B*. *cinerea* on tomato.

## Materials and methods

### Plant materials and chemicals

The work was carried out using tomatoes (*Lycopersicon esculentum* Mill.) (cv. Laifen No.1). The seedlings were friendly presented by Yifeng agricultural high-tech garden. In vitro assays, plants were cultured in pots in growth chambers with a 16 h light (90 μmol photons s^–1^ m^-2^)/8 h dark photoperiod and at a constant temperature of 22°C. In vivo assays, plants were planted in green house under normal conditions. All the seedlings and tomato fruits used for experiments were healthy and uniform. They had not been exposed to any pathogens. The commercial fungicide Methylthio diethofencarb wettable powder (thiophanate-methyl: 52.5%, diethofencarb: 12.5%) (Shandong Aovit Pesticide Co. Ltd, China) was used as the positive control.

### The causal pathogen of *B*. *cinerea*

*B*. *cinerea* isolate BcHY05 isolated from diseased tomato fruit with typical gray mold symptoms was used. Virulence of BcHY05 on tomato leaves was previously confirmed by us. In this work, BcHY05 was incubated on potato dextrose agar (PDA) in a growth chamber at 23°C for 8 d in order to collect conidia. The conidia were suspended with sterile distilled water containing 0.1% v/v Tween 80 and then filtered through four layers of sterile cheesecloth.

### Isolation and identification of *Pseudomonas* strain QBA5

To isolate the bacteria strains on the leaf surface, the discs of healthy leaves of tomato plants were immersed into the sterile distilled water (0.5 mL cm^-2^) in a 50-mL tube, and then they were vortexed for 10 min. The supernatant was diluted in a 10^−1^, 10^−2^, and 10^−3^ series and finally spread on a Luria Broth (LB) solid medium, which was incubated at 28°C for 24 h.

To identify the bacterial strain, the morphological characteristics and motility were observed using an optical microscope. Biochemical tests were performed according to Dong & Cai [34]. For molecular detection, the bacterium strain was inoculated into 3 mL of LB liquid medium with shaking at 28°C overnight. DNA extractions were done using a DNA preparation kit (TIANGEN) following the manufacturer’s standard protocol. 16S rDNA was amplified by standard PCR using universal primers 27f (5’-AGAGTTTGATCCTGGCTCAG-3’) and 1492r (5’- TACGGCTACCTTGTTACGACTT -3’). The resulting PCR product was purified with a Gel DNA Extraction kit (TIANGEN) and directly used for sequencing. The DNA sequence was finally analyzed using the NCBI BLAST (https://blast.ncbi.nlm.nih.gov/Blast.cgi) and was deposited in the GenBank.

### Preparation of the cell-free supernatant of QBA5 and its *in vitro* assay on *B*. *cinerea*

To obtain the cell-free supernatant, the QBA5 was incubated in a 100 mL flask containing 15 mL YEPD (yeast extract 3 g, peptone 10 g, dextrose 20 g per liter) liquid medium at 40°C while shaking at 130 rpm for 3 d. The culture was harvested and centrifuged at room temperature to collect the supernatant. Then the resulting supernatant was filter sterilized (0.22 μm pore diameter, Nantong Filterbio Membrane Co., Ltd, China).

For *in vitro* assay on *B*. *cinerea*, the inhibitory effects of the cell-free supernatant of QBA5 on conidia germination and germ tube elongation of *B*. *cinerea* were checked following the methods described by Wang et al. [3]. Conidia germination was verified if the length of germ tube was equal to or more than the conidia [35]. The conidia of *B*. *cinerea* were incubated in 100%, 75%, 50%, 25% and 10% cell-free supernatant at 23°C while shaking at 130 rpm, respectively. As a negative control, the conidia were incubated in YEPD. For each treatment, at least 200 conidia were examined microscopically for germination rate and germ tube length when at least 90% of the conidia in the YEPD control germinated. In each treatment, the final concentration of the conidia was adjusted to 5 × 10^5^ conidia mL^-1^. The inhibitory effect on mycelial growth was performed as described by Wang et al. [36]. Briefly, 3 mycelial disks of *B*. *cinerea* with a 0.5 cm diameter were incubated in 100%, 50% and 10% cell-free supernatant at 23°C while shaking at 130 rpm, respectively. After 10 d of incubation, the mycelia were harvested and dried. The inhibition rate of mycelial growth was calculated based on the formula [inhibition rate = (the mycelial weight of the control—the mycelial weight of treatment) / (the mycelial weight of the control) × 100%]. The colony plugs incubated in YEPD served as the negative control. Simultaneously, the inhibition rate resulting from Methylthio diethofencarb was also performed as the positive control. Methylthio diethofencarb was diluted according to the manufacturer’s instructions (final concentration: 667 mg L^-1^). Each trial was established for 3 replicates. And the results were the mean ± SE (standard error) of 3 independent experiments.

### Measurement of plasma membrane integrity

The conidia of the *B*. *cinerea* (final concentration: 5 × 10^5^ conidia mL^-1^) were incubated in undiluted cell-free supernatant of QBA5 at 23°C without shaking. As the negative control, the conidia were incubated in sterile distilled water. After 0, 2, 4, 6 and 8 h incubation, conidia samples were centrifuged at 8000 × g, and then staining and observations of conidia pellets were performed as described [3, 11, 37]. Briefly, the conidia were stained in 10 μg mL^-1^ propidium iodide (PI) for 5 min at 30°C and then centrifuged. The pellets were washed twice with the sodium phosphate buffer (pH 7.0). Observations were performed with a Zeiss Axioskop 40 microscope (Carl Zeiss, Oberkochen, Germany) equipped with an rhodamine filter set (Zeiss no. 15: excitation BP 546/12 nm, emission LP 590 nm). Three fields of view from each slide (at least 200 conidia) were chosen randomly, and the number of conidia in bright-field was defined as the total number. Membrane integrity (MI) was calculated according to the formula [MI = (the No. of total conidia—No. of stained conidia) / (the No. of total conidia) × 100%].

### Effect of cell-free supernatant of QBA5 on *Botrytis* infection of detached tomato leaves

Detached leaves from six-week-old tomato plants (cv. Laifen No.1) without any wounds and lesions were selected and surface-sterilized by soaking in a 2% aqueous sodium hypochlorite for 3 min. These leaves were then thoroughly rinsed with sterile distilled water, dried and placed into Petri dishes containing water-soaked filter paper. For inoculation treatments, each tomato leaf was divided into two parts along the vein: one part was sprayed on the leaf surface evenly with undiluted cell-free supernatant of QBA5, and the other part was sprayed with YEPD liquid medium as the negative control or sprayed with Methylthio diethofencarb as the positive control. Each treatment consisted of nine detached leaves. As soon as the surface of detached leaves dried, an 8 μL conidia suspension of *B*. *cinerea* with the final concentration of 2 × 10^5^ conidia mL^-1^ in 0.1% Tween 80 containing 0.5 mg mL^-1^ of glucose and 0.5 mg mL^-1^ of KH_2_PO_4_ [3] was dropped for the inoculation. All detached leaves were maintained at 23°C in the dark. After three days, the disease severity was assessed. The index of disease severity was rated on a scale of 0–4 (0: no disease symptom, 1: 0.1–5%, 2: 5.1–20%, 3: 20.1–40%, 4: 40.1–100%) as described by Lee et al. [38]. The value of disease severity was calculated using the following formula: Disease index (DI) = ((∑ (the number of diseased leaves × disease severity index) / (4 × the number of leaves rated)) × 100. The efficacy of QBA5 was calculated according to Abbott Formula [% effectiveness = (C − T) / C × 100], wherein C refers to DI of the negative control, and T refers to DI of the relevant treatment [3]. The results were the mean ± SE of three independent experiments.

### Effect of cell-free supernatant of QBA5 on *Botrytis* infection of ripe tomato fruit

In this experiment, the ripe tomato fruit (cv. Laifen No.1) was infected as described by Liu et al. [11]. Each fruit was wounded (3 mm deep and 3 mm wide) with a sterile nail at the equator. Then the same volume (15 μL) of undiluted cell-free supernatant of QBA5, Methylthio diethofencarb WP (wettable powder) as the positive control and YEPD liquid medium as the negative control, was put into each wound, respectively. After 4 h, a 6 μL conidia suspension of *B*. *cinerea* with the concentration of 2×10^5^ conidia mL^-1^ was added to each wound. Then all samples were kept in 0.2 m × 0.13 m × 0.15 m plastic boxes with sterile water to maintain a high humidity at 23°C in the dark for three days. The necrotic lesion on the tomato fruit around the wound was measured following Zhang et al. [39]. Each treatment consisted of 10 fruits, and the experiment was repeated for three times.

### Effect of cell-free supernatant of QBA5 on gray mold in tomato plant

Six-week old tomato plants of cv. Laifen No.1 planted in green house were used in this experiment. The plants were sprayed with the undiluted cell-free supernatant of QBA5 4h before and after the pathogen inoculation in the preventive and curative activity examination, respectively. The plants sprayed with liquid YEPD served as negative control, and plants sprayed with Methylthio diethofencarb WP served as positive control. For inoculation in all treatments, conidia suspension of *B*. *cinerea* with the final concentration of 5 × 10^5^ conidia mL^-1^ in 0.1% Tween 80 containing 0.5 mg mL^-1^ of glucose and 0.5 mg mL^-1^ of KH_2_PO_4_ was used. Every treatment consists of 10 plants and repeated 3 times. The results were evaluated 10 d after inoculation. The DI of the gray mold and the efficacy of QBA5 were calculated as above.

### Extraction and purification of the bioactive compounds of QBA5

QBA5 was cultured in a 1 L flask containing 500 mL YEPD liquid midium at 40°C with shaking at 130 rpm for 3 days. The supernatant was collected as mentioned above and extracted three times with an equal volume of chloroform. Then the organic phase was dried by rotary evaporation. And the resulting residue was dissolved in 20% methanol and passed through a 0.22 μm filter. To separate the active compounds, preparative high performance liquid chromatography (PHPLC) was conducted on a Waters 2545 series instrument (Waters, USA) equipped with a OBD-C18 column (19 mm ×150 mm, 10 μm, Waters, USA). The mobile phase used for PHPLC was methanol/H_2_O (1:4, v/v), applied with a flow rate of 8 mL/min, and ultraviolet detection was a 210 nm. The active fractions were collected based on the ultraviolet detection.

### In vitro assay of the bioactive compounds of QBA5 on conidia germination

To confirm the antifungal activity of the separated compounds of QBA5, the inhibition effect of the compounds on conidia germination were examined. In this assay, the conidia of *B*. *cinerea* were incubated in 100%, 75% and 10% of each compound separated by PHPLC. The conidia incubation and germination rate calculation were performed as above in this text.

### Statistical analysis

The data presented are the means of three independent experiments (means ± SE). All the statistical analyses were conducted using SPSS version 16.0 (SPSS InC., Chicago, IL, USA). Analysis of variance (ANOVA) was carried out to determine the effects of the treatments, and those means were compared by Duncan’s multiple range tests.

## Results

### Isolation and identification of *Pseudomonas* strain QBA5

In total, 68 bacterial strains were isolated from the healthy tomato leaves, among which one strain was selected for the subsequent experiments based on its inhibitory activity on conidia germination. This strain was termed as QBA5. To identify the strain, we carried out morphological, biochemical and DNA homologous analyses. The results revealed that QBA5 was negative Gram and may be belonged to the genus *Pseudomonas* based on its similarity in slight yellow colony, rod shape and mobility et al. (shown in Table 1). DNA homologous analysis showed that 16S rDNA of QBA5 (accession No. SUB2991790 QBA5 MF782453) shared 99% identity with a *Pseudomonas* clone YJQ-12 (accession number AY569288.1) in the GenBank database. Overall, the results would thus indicate that QBA5, a promising antifungal agent, was successfully isolated and identified to be a member of *Pseudomonas*.

**Table 1:** Morphological and biochemical characteristics of the bacterial stain QBA5.

Characteristics	QBA5
Color	Light yellow on LB medium
Shape	Rod
Motility	+
Gram	−
Arginine dihydrolase	−
V-P test	−
Glucose fermentation	+
Urease	−
Lipase	+
Benzazole test	−
Denitrification	−
Gelatin liquefaction	+
Catalase	−
Amylohydrolysis	−
Fluorescence	−
Nitrate reduction	−
Methyl Red	−

### Cell-free supernatant of QBA5 significantly inhibited conidia germination and germ tube growth of *B*. *cinerea*

**Fig 1:**
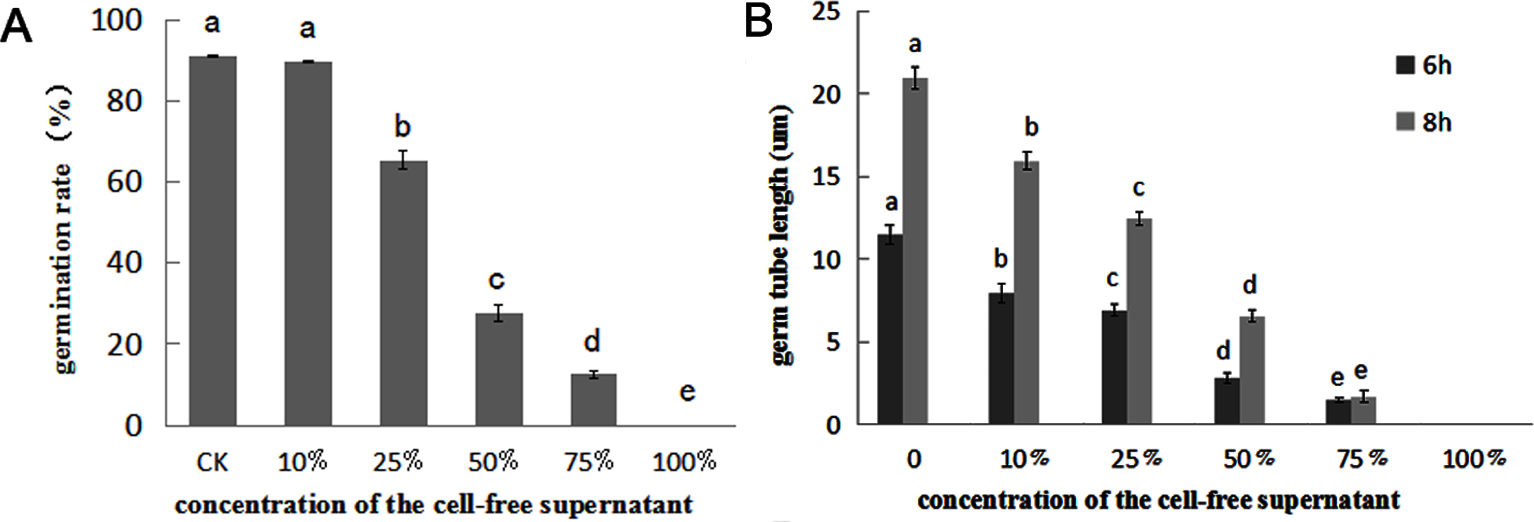
Cell-free supernatant of QBA5 significantly inhibited conidia germination and germ tube growth of *B*. *cinerea*. A: The germination rate of *B*. *cinerea* conidia after 8 h incubation with different concentrations of the cell-free supernatant of QBA5 at 23°C. B: Germ tube growth of *B*. *cinerea* at two time points after incubation with different concentrations of the cell-free supernatant of QBA5 at 23°C. Columns with different letters are significantly different. (*p*<0.01).

To evaluate antifungal efficiency of QBA5, we carried out conidia germination and germ tube growth of *B*. *cinerea* with the cell-free supernatant of QBA5. As shown in Fig 1A, the conidia germination was significantly inhibited, especially when the concentration of cell-free supernatant is more than 50% (*p*<0.01). After incubation for 8 h, the conidia germination rate was over 90% in the control. However, no conidium was detected to germinate in the 100% cell-free supernatant (Fig 1B). Furthermore, the inhibitory effect was positively related to the concentration of the cell-free supernatant. The results of the assays presented here indicate that the cell-free supernatant of QBA5 plays an important role in inhibition of conidia germination and germ tube growth of *B*. *cinerea*.

### The cell-free supernatant of QBA5 significantly inhibited the mycelial growth of *B*. *cinerea*

To further evaluate antifungal efficiency, we also examined the mycelial dry weight of *B*. *cinerea* incubated in the cell-free supernatant of QBA5 for 10 d. As shown in Table 2, the dry weight of the mycelia was remarkably decreased by the cell-free supernatant of QBA5 (*p*<0.01) which indicated that the mycelial growth of *B*. *cinerea* was significantly inhibited. Moreover, the inhibitory effect was positively related to the concentration of the cell-free supernatant. There is no significant difference in the inhibitory rate between the cell-free supernatant and the Methylthio diethofencarb WP (*p*<0.01). The result indicates that the cell-free supernatant of QBA5 could serve as an efficient alternative to Methylthio diethofencarb in controlling the mycelial growth of *B*. *cinerea*.

**Table 2:** Inhibitory effect of the cell-free supernatant of QBA5 on the mycelial growth of *B*. *cinerea* (*p*<0.01).

Treatment	Average dry weight (g)	Inhibition (%)
YEPD	0.718±0.012 a	0
10% cell-free supernatant	0.501±0.106 d	30.8
50% cell-free supernatant	0.379±0.011 c	47.6
100% cell-free supernatant	0.021±0.003 b	97.03
Methylthio dirthofencarb (667mg/L)	0.017±0.002 b	97.68

### The cell-free supernatant of QBA5 destroyed plasma membrane integrity of *B*. *cinerea*

**Fig 2:**
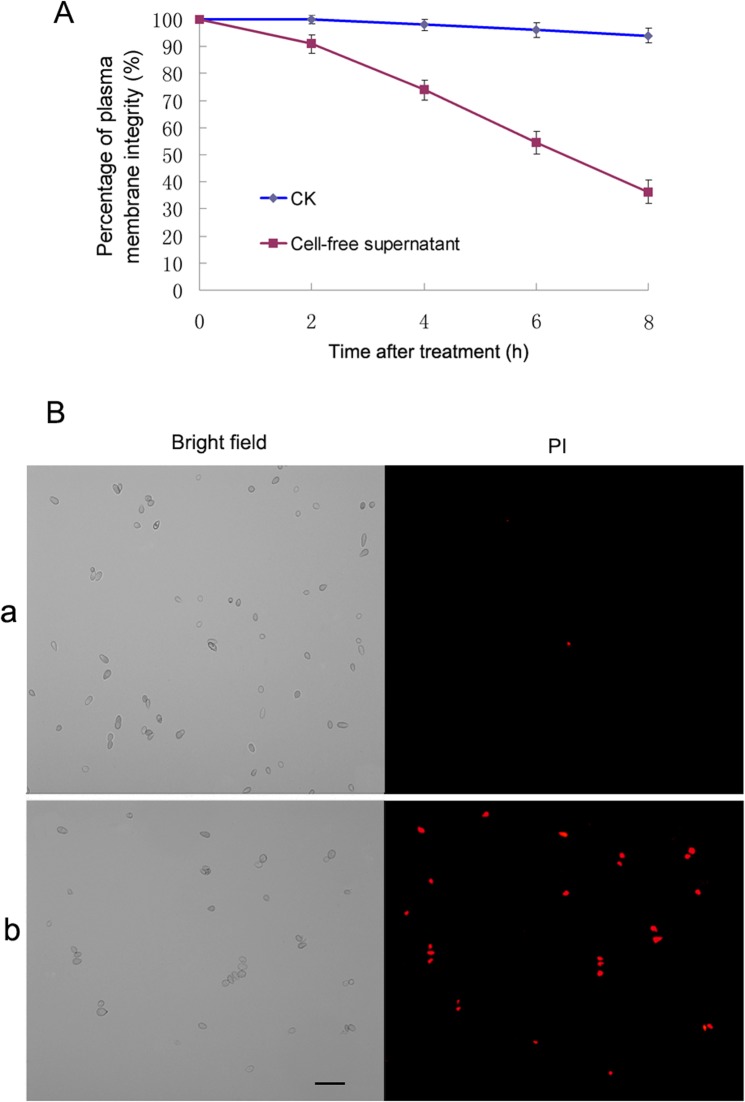
The cell-free supernatant of QBA5 destroyed plasma membrane integrity of *B*. *cinerea*. A: Percentage of plasma membrane integrity of *B*. *cinerea* conidia. B: Microscopy images of *B*. *cinerea* conidia after 8 h incubation and stained with propidium iodide (PI). The images on the left were captured under bright field, and the images on the right were captured under excitation light field. a: Conidia suspended in distilled water as the control. b: Conidia treated with cell-free supernatant of QBA5. Bar = 20μm.

**Fig 3:**
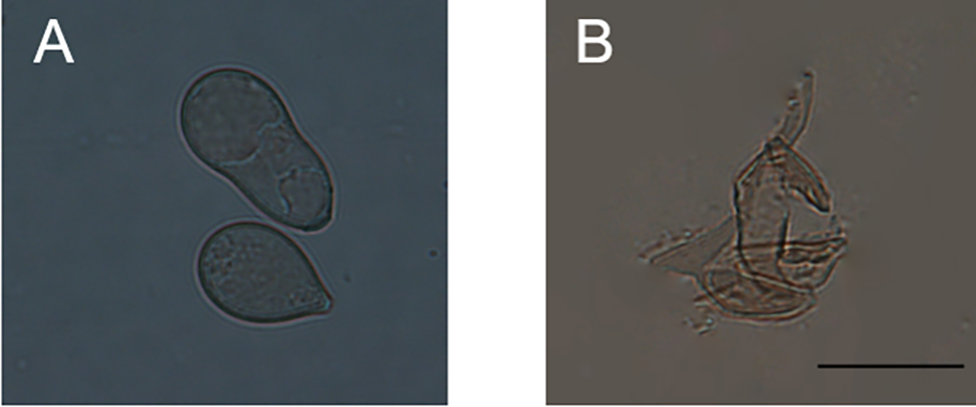
The cell-free supernatant of QBA5 had dramatic inhibitory effects on conidia of *Botrytis cinerea*. A: Conidia suspended in sterile distilled water as control. B: Conidia treated with the cell-free supernatant of QBA5 for 24 h. Bar = 10μm.

To investigate the underlying mechanisms by which the cell-free supernatant of QBA5 exhibited the inhibition to conidia germination and mycelial growth, we detected the plasma membrane integrity of *B*. *cinerea* conidia. As shown in Fig 2A, the plasma membrane integrity of *B*. *cinerea* conidia was seriously damaged by the cell-free supernatant of QBA5. Moreover, with elongation of the incubation time, the membrane integrity of *B*. *cinerea* conidia declined rapidly (*p*<0.05). The direct evidence was shown in Fig 2B, the red fluorescence emitted from conidia with destroyed plasma membranes was observed under the fluorescence microscope, while no fluorescence could be detected from conidia with intact plasma membranes. After incubation in the cell-free supernatant of QBA5 for 24 h, cell wall degradation of some conidia were observed (Fig 3). The data presented here indicate that the damage of the plasma membrane and the degradation of the cell wall might contribute together to the inhibiton of *B*. *cinerea* by supernatant of QBA5.

### Cell-free supernatant of QBA5 greatly inhibited disease severity not only on detached leaves but also on fruits

**Fig 4:**
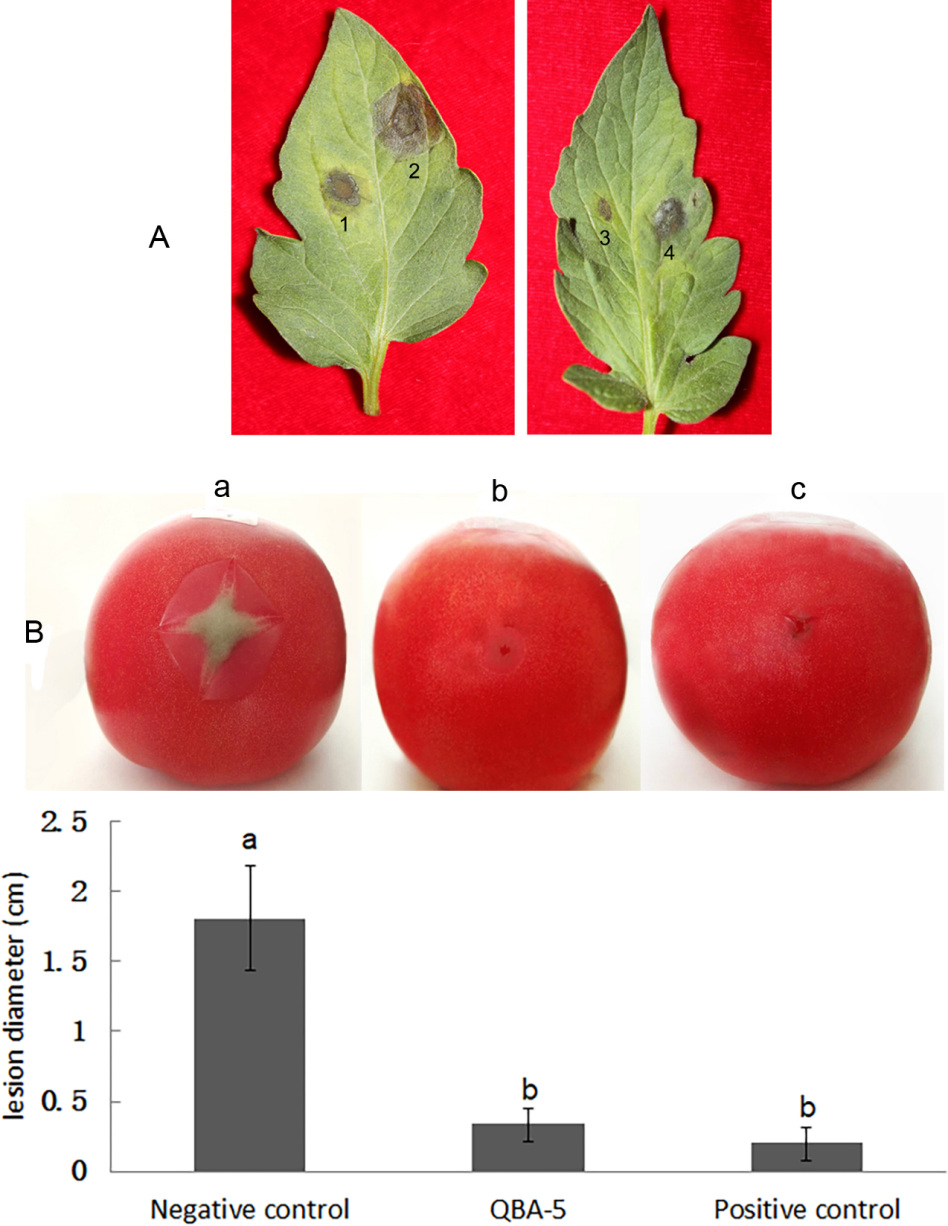
The cell-free supernatant of QBA5 greatly inhibited disease severity not only in detached leaves but also fruits. A: Disease severity of gray mold on tomato leaves. 1 and 4: leaves treated with cell-free supernatant of QBA5. 2: Leaves treated with YEPD as the negative control. 3: Leaves treated with Methylthio diethofencarb (667mg/L) as the positive control. B: Disease severity of gray mold on tomato fruits. a: Tomato fruit treated with YEPD as the negative control. b: Tomato fruit treated with the cell-free supernatant of QBA5. c: Tomato fruit treated with Methylthio diethofencarb (667mg/L) as the positive control. Columns with different letters are significantly different. (*p*<0.05).

As shown in Fig 4A, lesions caused by a *B*. *cinerea* infection on the detached tomato leaves were found in all treatments. However, there is significant difference in the disease severity between the leaves treated by supernatant of QBA5 and in the negative control (*p*<0.05). The supernatant of QBA5 exhibited an inhibitory effect on the gray mold (the disease index = 12.5, the effectiveness = 70.0%), whereas in the positive control, Methylthio diethofencarbit exhibited a much higher inhibitory effect than the supernatant of QBA5 (the disease index = 3.2, the effectiveness = 94.9%).

The results of the inoculation assays on tomato fruits showed that *B*. *cinerea* caused necrotic lesion on all tomato fruits in every treatment. Compared with the negative control, as showed in Fig 4B, the supernatant of QBA5 exhibited a strong inhibitory effect on the gray mold disease on tomato fruits. The infection of *B*. *cinerea* leads to just a little hygrophanous lesion around the inoculation point on tomato fruits which were pretreated with the supernatant of QBA5, the average diameter of lesions is 0.34 cm. There is no significant difference in the average diameter of necrotic lesions between the tomato fruits treated by the supernatant of QBA5 and Methylthio diethofencarbit (*p*<0.05). However, the infection of *B*. *cinerea* caused a large necrotic lesion with a thick layer of gray mycelia on the tomatoes in the negative control, the average diameter of lesions reaches 1.81 cm. The results of the inoculation demonstrated that supernatant of QBA5 has a very strong inhibition to *B*. *cinerea* on tomato plant and fruit.

### Cell-free supernatant of QBA5 exhibited higher effect on the gray mold on tomato plants in preventive treatment

To examine the inhibitory effect of QBA5 on the gray mold on tomato plants, the assay was arranged in a green house. As shown in Table 3, the DI is 39.34 and 56.96 on the tomato plants sprayed with cell-free supernatant of QBA5 in preventive and curative treatment, respectively. While the DI on the tomato plants in the negative control is 83.14. The result indicated that the development of gray mold on tomato plants was inhibited by cell-free supernatant of QBA5. The control effect of the cell-free supernatant of QBA5 is 52.68% in the preventive treatment while is 31.49% in the curative treatment. The result indicated that cell-free supernatant of QBA5 played a better inhibitory role on gray mold disease in a preventive manner.

**Table 3:** The preventive and curative effects of the QBA5 100% cell-free supernatant on the infection of *B*. *cinerea* on tomato plants.

Treatment	Preventive treatment DI control value (%)	Curative treatment DI control value (%)
gative control	83.14 0	83.14 0
100% cell-free supernatant	39.34 52.68	56.96 31.49
Positive control	26.42 68.22	22.56 72.86

Negative control: the tomato plants were sprayed with YEPD.

Positive control: the tomato plants were sprayed with Methylthio dirthofencarb (667mg/L).

### Four bioactive compounds with distinct antifungal activity were collected from the cell-free supernatant of QBA5

**Fig 5:**
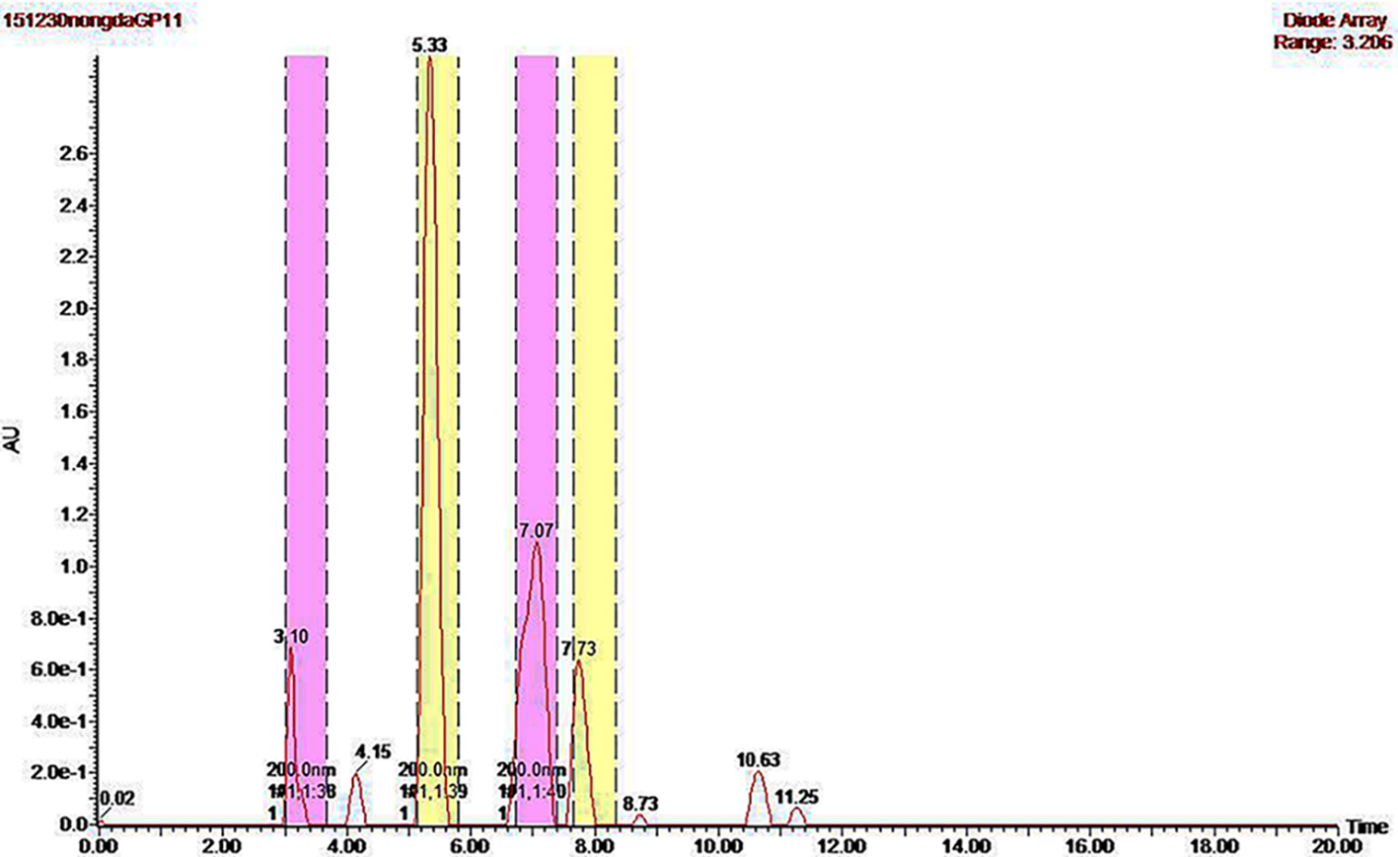
The bioactive compounds in the supernatant of QBA5 were separated with preparative high performance liquid chromatography (PHPLC). Four distinct eluent peaks were detected at 3.10 min, 5.33 min, 7.07 min and 7.73 min by their ultraviolet absorption at 210 nm.

**Fig 6:**
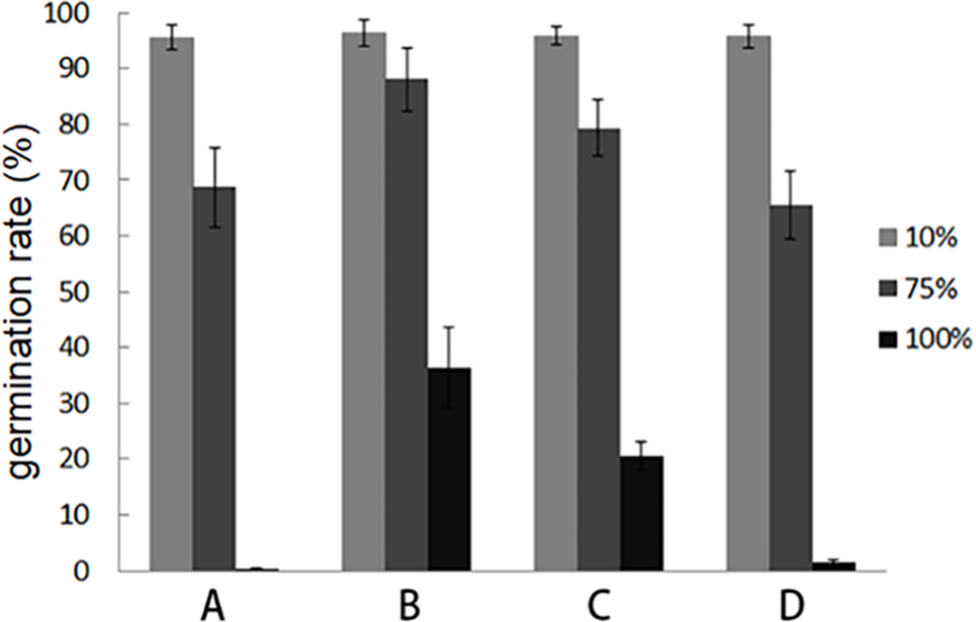
The inhibitory effect of the four active compounds on the conidia germination of *B*. *cinerea*. A, B, C, and D represent the compounds collected at 3.10 min, 5.33 min, 7.07 min and 7.73 min from the supernatant of QBA5 by preparative high performance liquid chromatography (PHPLC), respectively.

To separate the active components with antifungal activity in the supernatant of QBA5, the supernatant of QBA5 was extracted with chloroform. And the antifungal activity of the chloroform extracts was then assayed with *B*. *cinerea*. The result showed that conidia germination of the *B*. *cinerea* was significantly inhibited by the chloroform extracts which indicated that the bioactive compounds were mostly contained in the chloroform extracts. So, the bioactive compounds were further separated with PHPLC. As shown in Fig 5, four different compounds were collected based on the ultraviolet absorb peak occurred at 3.10 min, 5.33 min, 7.07 min and 7.73 min, respectively. The antifungal activity of the four compounds was examined. The results (Fig 6) showed that the four compounds have different inhibitory effects on the conidia germination. Furthermore, the plasma membrane integrity of the conidia treated by the compounds collected at 3.10 min and 7.73 min was detected. The results showed that the plasma membrane integrity of the conidia was severely damaged by the former compound but was not damage by the later compound. These results indicated that the inhibition of *B*. *cinerea* by QBA5 resulted from multiple bioactive compounds together with different mechanisms.

## Discussion

The phyllosphere is an important microorganism source of BCA for controlling plant diseases. With the aim to acquire some antagonists to control gray mold on tomato, in this study, we isolated the bacterial strains from healthy tomato leaves. As a result, we identified one strain belonging to the member of the genus *Pseudomonas* based on its morphological, biochemical characters and DNA sequence. The bacterial strain was termed as QBA5 exhibiting significant antifungal activity against *B*. *cinerea*. Compared with the liquid cultures of QBA5, in *in vitro* assays, the cell-free supernatant of QBA5 has almost the same inhibitory effect on conidia germination, germ tube elongation and mycelial growth of *B*. *cinerea*. This implies that QBA5 may play its inhibitory roles against *B*. *cinerea* by some secreted bioactive components. Therefore, in our experiments, the cell-free supernatant of QBA5 was used to examine the inhibitory effect and underlying mechanism of QBA5 against *B*. *cinerea*.

Antibiosis is a principal mechanism of biological control by which antagonists can kill or inhibit potential pathogens [40, 41]. It has been reported that some *Pseudomonas* spp. successfully achieved their biocontrol functions by producing some bioactive metabolites, such as lipopeptides, 2,4-diacetylphloroglucinol (DAPG) and phenazine-1-carboxylic acid (PCA) [42, 43, 44]. Based on the fluorescence from the conidia treated by PI in this study, it was believed that some bioactive metabolites produced by QBA5 played crucial roles in inhibiting *B*. *cinerea*. The bioactive metabolites destroyed the plasma membrane of conidia, and led to the entrance of PI into the cell. Cell wall-degrading enzymes (CWDEs) are important bioactive substances against fungal pathogens of some biological control bacterial species [45, 46, 47]. The cell wall degradation of the conidia treated by cell-free supernatant of QBA5 was also observed. It implied that some active degradation-related enzymes might be produced by QBA5 and make functions against *B*. *cinerea*. Taking into account the fact that the damage to integrity of plasma membrane occurred much earlier than the degradation of the cell wall, we proposed in our study that the injury of plasma membrane would be a primary mechanism of QBA5 to inhibit the *B*. *cinerea*. The discovery of the bioactive compound without activity to membrane damage makes a well interpretation to the difference between the effect on conidia germination (Fig 1) and plasma membrane integrity (Fig 2) of the supernatant. The result indicated that the inhibition to the conidia germination by the supernatant is a joint action of those different compounds.

In this study, the action mode of QBA5 against gray mold disease was also investigated. The significant inhibitory effect of QBA5 was observed when the tomato fruits and plants were treated with cell-free supernatant for 4 h before pathogen inoculation. However, if the pathogen inoculation was carried out for 4 h prior to treatment with the cell-free supernatant, the inhibitory effect was much lower both on tomato fruits and plants. Therefore, it suggested that QBA5 plays its biocontrol roles against gray mold in a preventive manner, and inhibition to the conidia germination should be the dominant role of QBA5 to control the gray mold caused by *B*. *cinerea*. The inoculation results showed that gray mold disease on tomato fruits and plants was significantly inhibited by QBA5, which indicates that QBA5 could be a considerably potential alternative for chemical fungicides in reducing the damage of gray mold disease.

## Conclusion

In this study, we isolated a novel *Pseudomonas* strain QBA5 which exhibited significantly antifungal activity through multiple bioactive compounds with distinct mechanism. Based on the inhibitory effect on the development of gray mold on tomato fruits and plants, the strain QBA5 might be a considerably potential to reduce the damage caused by gray mold disease on tomato in the future.

## Abbreviations

PHPLC: preparative high performance liquid chromatography
BCA: biological control agent
VOC: volatile organic compounds
PDA: potato dextrose agar
LB: Luria Broth
MI: Membrane integrity
DAPG: 2,4-diacetylphloroglucinol
PCA: phenazine-1-carboxylic acid
CWDE: cell wall-degrading enzyme
